# The effects of selenium supplementation on inflammatory markers in critically ill patients

**DOI:** 10.1007/s42452-022-05208-4

**Published:** 2022-11-08

**Authors:** Ata Mahmoodpoor, Elnaz Faramarzi, Anita Reyhanifard, Ali Shamekh, Saba Nikanfar, Akbar Azizi-Zeinalhajlou, Sarvin Sanaie

**Affiliations:** 1 Department of Anesthesiology, Faculty of Medicine, Tabriz University of Medical Sciences, Tabriz, Iran; 2 Liver and Gastrointestinal Diseases Research Center, Clinical Research Institute, Tabriz University of Medical Sciences, Tabriz, Iran; 3 Student Research Committee, Aging Research Institute, Tabriz University of Medical Sciences, Tabriz, Iran; 4 Department of Clinical Biochemistry and Laboratory Medicine, Faculty of Medicine, Tabriz University of Medical Sciences, Tabriz, Iran; 5 Research Center of Psychiatry and Behavioral Sciences, Aging Research Institute, Tabriz University of Medical Sciences, Tabriz, Iran; 6 Research Center for Integrative Medicine in Aging, Aging Research Institute, Tabriz University of Medical Sciences, Tabriz, Iran

**Keywords:** Critical illness, Inflammation, Oxidative stress, Selenium, Supplemtation

## Abstract

**Abstract:**

Low serum selenium (Se) levels have been shown in critical illness, which is associated with poor clinical outcomes and a higher mortality rate. Se plays an important role in inflammation and oxidative stress. Since the overproduction of inflammatory cytokines and increased oxidative stress is a major component of critical illnesses, its supplementation has been demonstrated to have promising effects on critically ill patients. This study aims to review the evidence regarding the effects of Se supplementation on inflammatory and oxidative markers in critically ill patients. The literature review highlights alterations of inflammatory markers, including procalcitonin, leukocyte count, albumin, prealbumin, C-reactive protein (CRP), inflammatory cytokines, and cholesterol following Se supplementation in critically ill patients. Besides, the antioxidant properties of Se due to its presence in the structure of several selenoenzymes have been reported.

**Article highlights:**

- Low serum Se level have been shown in critical illness, which is associated with poor clinical outcome and higher mortality rate.
- Se plays an important role in inflammation and oxidative stress.
- Se supplementation can have promising effects by alterations of inflammatory markers and its antioxidant properties for critically ill patients.

## Introduction

Selenium (Se) is an essential trace element that plays an important role in the immune system through redox balancing, anti-inflammatory, and antioxidant activities [1]. By incorporating into selenoproteins, selenocysteine has a profound effect in reducing inflammation and oxidative stress. Selenoenzymes such as five glutathione peroxidases (GSH-Px), methionine sulfoxide reductase 2, and three thioredoxin reductases (TrxR) contain selenocysteine at their active site. The selenoenzymes inhibit proinflammatory cell metabolisms and protect cell components against oxidation [2]. The organoselenium compound which has been most studied for its anti-inflammatory activity is diphenyl diselenide through its ability to modulate macrophage activation and inhibit the production of NO [3]. Moreover, selenium nanoparticles (nanoselenium) are a novel elemental form of selenium which have been reported to have more bioavailability and beneficial bioactivities. Anti-oxidant effect exerted by nanoselenium is mediated through improvement of GPx, superoxide dismutase (SOD) and catalase (CAT) activities, as well as direct free radicals scavenging activity. Moreover, inhibition of lipid peroxidation by the decrement of TBAR has been reported by nanoselenium [4]. Therefore, Se supplementation is a promising adjunctive therapy in patients with critical illnesses, including sepsis, systemic inflammatory response syndrome (SIRS), and COVID-19, a rapidly emerging pandemic [5]. Critical illness is defined as a condition of oxidative stress, hyper-inflammation, and impaired mitochondrial or immune system function.[6] A growing body of evidence suggests that oxidative stress contributes to the development of critical illness complications, including multiple organ failure and SIRS [7]. Reactive oxygen species (ROS) play an essential role in linking inflammation and oxidative stress. In this sense, the production of ROS as a result of the inflammatory response can promote oxidative stress, which, in turn, can cause inflammation by inducing pro-inflammatory factors [8].

Critically ill patients are at increased risk of micronutrient deficiency due to the alteration of micronutrient levels in the plasma, which may be resulted from the reduced levels of their carrier proteins [9]. In this regard, decreased levels of Se have been reported in septic, SIRS, and coronavirus disease 2019 (COVID-19) patients, especially those with a critical illness who receive poor-quality diets [10, 11]. Considering the anti-inflammatory and antioxidant properties of Se, it can be considered an attractive therapeutic strategy in relieving inflammation-related conditions in critically ill patients. However, the exact mechanism is not clearly understood. In the present article, we review the most current evidence regarding the effects of Se supplementation on inflammatory and oxidative markers in critically ill patients.

## Se in critically ill conditions

During critical illness, the levels of micronutrients are modified. In this regard, redistribution of trace elements from circulation to the tissues, which have a crucial role in the proliferation of immune cells and protein synthesis, is observed in SIRS. The decrease in the trace element carrier proteins may consider as another underlying reason which alters the micronutrient levels [12]. Previously, low Se plasma levels were reported in intensive care unit (ICU) patients [13]. In another study, the patients with multiorgan failure or septic shock revealed lower levels of Se and selenoprotein P on ICU admission [14]. These agree with Sakr et al. findings in which Se levels presented a descending trend during the ICU stay in SIRS patients or those with organ failure [15]. The negative correlation of Se levels in patients with SIRS with sepsis severity scores has been demonstrated. In that research, Se levels lower than 0.70 µmol/L showed an association with higher rates of mortality and organ failure [16]. Moreover, the decreased levels of Se seem to be common in COVID-19, especially in those with severe disease. Se deficiency was associated with a higher risk of mortality in patients with COVID-19 [11]. Due to the observed correlation of Se status with clinical outcomes, it seems that early assessment of its levels on ICU admission may be a valuable predictor of survival in critically ill patients [17].

## Se supplementation in critically ill conditions

Concerning the antioxidative and anti-inflammatory role of Se, promising findings have been reported with Se supplementation in critical illness. In this regard, over the past 20 years, several clinical trials demonstrated the benefits of Se therapy, especially intravenous bolus administration, on clinical outcomes in patients with a critical illness [18]. In recent years, the effects of seleno-compounds have been evaluated in ICU patients, particularly those with systemic inflammation, sepsis, and severe sepsis [18]. Se supplementation in critically ill patients has been reported to lower mortality rates, organ failure, and infections compared to the control group [18, 19]. Furthermore, the improving effects of Se supplementation on immune response and its role in lowering the risk of SARS-CoV-2 infection has been reported [20]. The route of Se delivery, the dose of Se (high or low dose), bolus or continuous administration, and the patient selection are different between studies and may influence the observed effects of supplementation [6, 21, 22]. In this regard, it has been demonstrated that parenteral substitution of Se reduced mortality and infection rate in sepsis syndromes [18]. Nevertheless, intravenously administered Se at a high dose has demonstrated primary pro-oxidant activity, which may be helpful in the early stages of the disease [18]. Furthermore, due to the lack of sufficient understanding about the role of Se therapy in renal failure, the high-dose Se should be used with caution in ICU patients with renal failure [23]. The beneficial effects of Se supplementation may stem from its presence in the structure of several selenoenzymes. These selenoenzymes show various antioxidant and immunomodulatory properties since they are involved in redox signaling, antioxidant defense, and immune responses [17]. However, the exact mechanism for the beneficial effects of Se supplementation in critically ill patients is not completely understood.

## Se supplementation and inflammatory markers

**Fig. 1:**
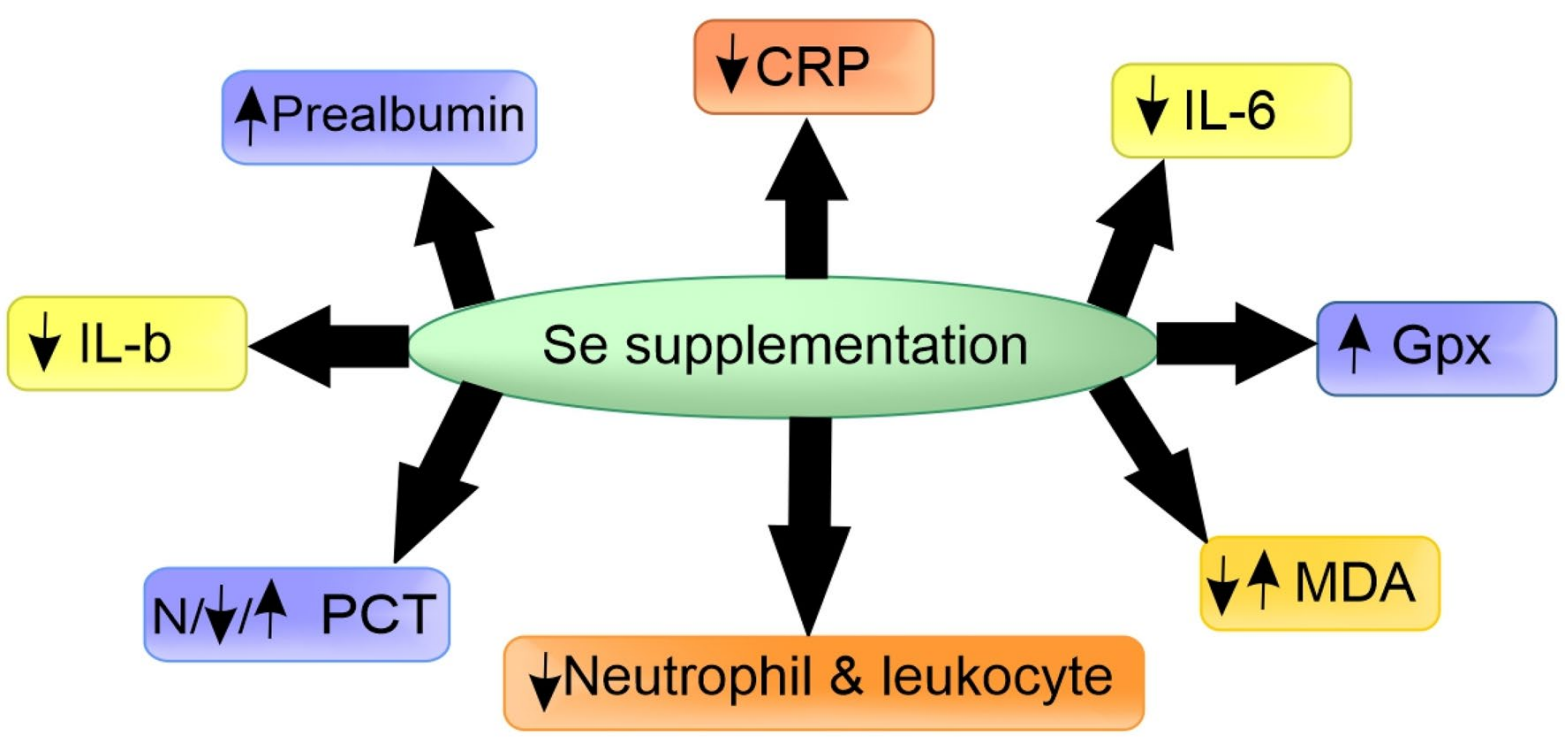
Schematic view illustrating the effects of Selenium supplementation on inflammatory markers in critically ill patients

A large body of research has demonstrated a close association between Se deficiency and inflammation and regulatory effects of Se on inflammation via affecting the expression of various cytokines [24]. In this section, we performed a review of the literature regarding the effects of Se supplementation on inflammatory markers, including procalcitonin, leukocyte count, albumin, prealbumin, CRP, inflammatory cytokines, and cholesterol in critically ill patients (Fig. 1).

### Procalcitonin

Procalcitonin (PCT), a prohormone of calcitonin, is considered as one of the inflammatory markers in discriminating sepsis from other causes of SIRS that are not related to infectious [25]. The cutoff value of PCT for diagnosing sepsis or septic shock was 2 ng/ml [26]. Critically ill patients have elevated concentrations of PCT, as observed in individuals with organ failure, SIRS, and infection [15]. A large number of papers showed the inverse correlation between plasma Se concentrations and PCT levels [15, 27]. High-dose Se supplementation in sepsis, a major cause of death in critically ill patients, resulted in a faster decrease in PCT levels between baseline and days 7 and 14 [27] (Table 1). Moreover, Se therapy (starting with 1000 µg on the first day and continuing to 200 µg after one week) decreased PCT levels at day 10 in patients with SIRS/ sepsis [28]. In contrast, Sakr et al. [29] indicated elevated levels of PCT in severe sepsis after receiving 1000 µg of sodium selenite intravenously. Due to a lack of information regarding the function of organs at the onset of severe sepsis, sodium selenite may reveal its pro-oxidant properties at high doses [30]. However, Woth et al. [31] did not report a significant change in PCT values following sodium selenite treatment (1000 µg/2 h) in severe sepsis patients.

**Table 1:** Effects of Se supplementation on inflammatory and oxidative markers in critically ill patients

Subjects	Sample size (N)	Study design	Se supplementation	Outcomes	References
Critically ill patient	24	RCT	Parenteral sodium selenite for 3 weeks (1st week: twice 5oo µg/day, 2nd week: once 500 µg/day, 3rd week: thrice-daily 100 µg)	↓ MDA	[71]
Critically ill patient	100	Non-randomized clinical trial	500 µg Se twice daily infused over 2 h for 5 days	↓ CRP	[52]
End-stage chronic renal failure patients	53	Comparative study	200 µg daily for 3 months as Se-enriched yeast	↑ GSH-PX3 (at the primary stage of the disease)	[87]
Septic ICU patients	40	RCT	A continuous IV infusion of sodium selenite: 474, 316, and 158 µg (high dose) for 3 days each, and thereafter 31.6 µg/day (standard dose)	No change in F2-isoP levels ↑ GSH-PX3	[49]
Patients with severe SIRS, sepsis, and septic shock	238	Clinical trial	IV sodium selenite: 1000 µg/30 min followed by 1000 µg/day continuously for 14 days	↑ GSH-PX3	[82]
Septic patients	68	A prospective, randomized, double-blind study	Infusion of 1000 µg sodium selenite on the 1st day, 500 µg 2nd day and 200 µg on the following days	↓ PCT	[28]
Sheep model of sepsis	21	Experimental study	Bolus injection (2 mg sodium selenite, followed by 0.06 µg/kg. h) or continuous infusion (4 µg Se per kg.hr)	↓ IL-6 (bolus vs. control)	[58]
SIRS or sepsis patients	150	RCT	1000 µg sodium selenite pentahydrate (Na2SeO3.5H2O) on 1st day, 500 µg daily on days 2–14	↓ PCT (day 7 and 14 vs. baseline) ↑ prealbumin No change in albumin ↓ CRP (day 7 and 14 vs. baseline but no difference between Se-supplemented and control groups) ↑ cholesterol ↑ GSH-PX3	[27]
SIRS patients	40	Clinical trial	A bolus IV injection of 2000 µg selenite followed by continuous infusion of 1600 µg Se/day for 10 days	↑ GSH-PX3 (day 3 and 7 vs. baseline)	[90]
Septic shock with acute organ failure	–	Case report	IV injection of 750 µg/day Se over 6 days + 2 g/day glutamine	↓ PCT ↓ neutrophils and leukocyte count	[38]
Septic patients	72	Observational study	A continuous infusion of sodium selenite at 750 µg/day for 6 days	↑ GSH-PX3	[86]
Severe sepsis	1040	Retrospective study	A bolus IV injection of 1000 µg Na2SeO3.5H2O for 30 min, followed by infusion of 1000 µg per day for 14 days	↑ PCT	[29]
Severe sepsis	40	RCT	1000 µg/2 hr sodium selenite	No change in PCT, CAT, and SOD levels ↑ MDA	[31]
Septic patients	54	RCT	A bolus IV injection of 2 mg sodium selenite followed by 1.5 mg continuous infusion for 14 days	No change in IL-6 ↑ GSH-PX3	[57]
Mechanically ventilated critically ill patients	99	RCT	3000 µg on the 1st day and 1500 µg on the following 9 days	↑ GSH-PX3	[85]
Septic patients	54	RCT	High-dose parenteral sodium selenite (2 mg bolus followed by 1.5 mg continuous intravenous infusion daily for 14 days)	No change in SOD levels	[92]
ARDS patients	40	RCT	4 mg IV sodium selenite at 1st day, 1 mg/12 h for 3 subsequent days and 1 mg/day for additional 6 days	↓ CRP and IL-6 during the study period but no difference between Se-supplemented and control groups IL-beta: Decreasing trend in Se-supplemented group	[51]

*ARDS* Acute respiratory distress; *CRP* C-reactive protein; *CAT* catalase; *F2-isoP* F2-isoprostanes; *GSH-PX-3* Glutathione peroxidase 3; *IV* Intravenous; *MDA* Malondialdehyde; *PCT* procalcitonin; *RCT* Randomized clinical trial; *SIRS* Systemic inflammatory response syndrome; syndrome; *SOD* Superoxide dismutase

### Leukocyte count

It has been demonstrated that total leukocyte count can be used as a diagnostic marker for bacteremia in critical illnesses and its high levels were observed in conditions, including SIRS, infection, and organ failure [32]. However, for better prediction of bacteremia, the neutrophil/lymphocyte count ratio along with lymphocytopenia have been suggested [33]. Accumulation of leukocytes in the microvasculature, which prevents reperfusion, deteriorates tissue damage by producing ROS [34]. The severe conditions, including SIRS or sepsis, can consume the Se content of leukocytes, resulting in lower Se levels in these patients [35]. In this regard, a negative correlation has been reported between the minimum plasma levels of Se and maximum leukocyte count in ICU patients [15]. The apoptosis-inducing effects of supplementation with high doses of Se on leukocytes have been demonstrated in previous studies, which can be mediated by nuclear factor kappa B (NF-kB) inhibition [36]. Furthermore, it has been demonstrated that long-term administration of sodium selenite decreases the leukocyte and neutrophil count in circulation [37]. In addition, administration of Se intravenously as adjuvant therapy (750 µg/day over six days) decreased neutrophils and leukocyte count in patients with acute organ failure [38].

### Albumin and prealbumin

Albumin and its precursor prealbumin (also called transthyretin) are considered negative acute-phase proteins. Their concentrations may represent inflammation and risk of mortality more than nutritional status in critically ill patients In this regard, the inhibitory effects of inflammatory cytokines on the prealbumin synthesis and inverse correlation of serum albumin and prealbumin levels with inflammatory biomarkers of C-reactive protein (CRP) and neutrophil-lymphocyte ratio (NLR) have been demonstrated in ICU patients [39]. The increased vascular permeability in critically ill patients may also result in decreased concentrations of prealbumin and albumin which subsequently alter the distribution of Se throughout the body [40]. Approximately 6–10% of Se is bound to albumin and the positive correlation of plasma Se concentration with albumin levels has been reported [15]. In this regard, patients with respiratory diseases who had lower serum levels of Se showed lower albumin than those who had normal levels of Se [41]. However, in a recent study, plasma Se was not correlated with albumin levels in critically ill patients [42]. In sepsis patients, while the Se levels were correlated with prealbumin in both standard and high-dose Se supplementation groups, the correlation with albumin was observed only in standard-dose Se-supplemented patients [43]. In another study by the same group, although high-dose Se supplementation in septic patients did not reveal any difference in albumin levels, prealbumin increased in the Se-supplemented group at days 7 and 14 compared to baseline levels. Moreover, dietary supplementation of rats with supraphysiological doses of Se increased transthyretin levels in plasma [44]. Therefore, Se supplementation may accelerate the restoration of prealbumin levels. The unchanged albumin levels following Se supplementation may stem from the effects of albumin administration or its long half-life [27].

### CRP

CRP is an inflammatory marker and a positive acute-phase protein. High levels of this protein are observed in critical illnesses, including organ failure, SIRS, and sepsis [15]. The alteration in CRP concentrations is associated with sepsis prognosis [45]. Since the high levels of CRP during the first days of infection are decreased following treatment or regression of inflammation, it can be used for treatment efficacy monitoring [46]. Chronic and acute inflammatory conditions with higher CRP values showed lower Se levels [47]. In this regard, an inverse correlation has been observed between the minimum concentrations of Se and maximum CRP in the serum [15]. Similar results have been reported by Iglesias et al. in critically ill children and also by other researchers in septic patients [27, 48]. However, some studies have reported no correlation between Se and CRP in both control and Se-supplemented groups, and a negative correlation was observed only on admission day [42, 49]. By increasing selenoprotein synthesis and thereby suppressing CRP production, Se supplementation can compensate for lower levels of Se in serum and liver, and as a result, inflammation will be attenuated [50] Although Se supplementation with sodium selenite decreased CRP levels in acute respiratory distress syndrome (ARDS) patients during the study period, the differences between Se-supplemented and control groups were not significant [51]. Valenta et al. [27] showed that CRP levels were decreased between baseline and day 14 in Se supplemented septic patients. Nevertheless, there were no significant differences in CRP concentrations between Se-supplemented and control groups. However, the plasma levels of CRP reduced significantly in critically ill patients who received Se for 5 days [52]. More detailed studies are needed to confirm the effect of Se supplementation on CRP values.

### Inflammatory cytokines

Interleukin-6 (IL-6), an inflammatory cytokine, is elevated in the plasma during ICU hospitalization in patients with severe sepsis. It has been demonstrated that IL-6 serum levels are associated with the severity of organ dysfunction, mortality, or clinical outcomes in critical illness [15, 53]. In sepsis, decreased Se was associated with elevated levels of IL-6 [54]. Moreover, the inverse correlation of plasma Se concentration with serum IL-6 was reported in critically ill patients [15]. The same correlation was also found in cirrhotic and elderly patients [55, 56]. In critically ill patients, the effects of Se supplementation on IL-6 concentrations have been a source of controversy. Supplementation with sodium selenite decreased serum levels of IL-6 in critically ill patients with ARDS compared to their baseline values. However, this reduction was not significantly different from the control group and did not reveal any benefit of Se supplementation in the downregulation of this cytokine in plasma [51]. In agreement with the mentioned findings, Chelkeba et al. [57] did not report the influence of supplementation with Se on IL-6 levels in patients with sepsis admitted to ICU. Nevertheless, using a sheep model of sepsis, a group of researchers reported that a bolus injection of sodium selenite significantly decreased IL-6 levels compared to the control group [58]. Although it seems that Se supplementation may improve inflammatory conditions such as severe sepsis or septic shock, further work is required to establish this. IL-1b is another pro-inflammatory cytokine that plays a key role in acute and chronic inflammatory disorders. It has been demonstrated that serum concentrations of Se are inversely correlated with IL-1b in critically ill patients [51]. Besides, while IL-1b was lower in critically ill patients supplemented with Se, the serum levels of this cytokine were similar on the last day of treatment [51]. Thus, it seems that supplementation with this micronutrient may not be helpful in reducing IL-1b values. Furthermore, supplementation with high dose Se did not affect the IL-8 levels in the plasma of patients with sepsis [57].

### Cholesterol

Alteration of lipid profile is one of the well-known metabolic changes observed in critically ill patients, particularly those with sepsis. The pro-inflammatory cytokines cause hypertriglyceridemia and hypocholesterolemia by inducing adipose tissue lipolysis and fatty acid synthesis in the liver [59]. Due to the inverse correlation of total cholesterol levels with pro-inflammatory cytokines, hypocholesterolemia is proportional to the severity of disease in critical illness. Furthermore, cholesterol may serve as a marker for sepsis and can predict the outcome better than other biomarkers such as CRP or PCT [60, 61]. It has been demonstrated that in patients with SIRS/sepsis, Se levels are correlated with cholesterol. The same pattern was reported in patients supplemented with Se suggesting the possible role of Se supplementation in raising cholesterol levels and reducing its subsequent detrimental effects [43]. In this regard, the beneficial effects of Se supplementation on cholesterol levels were reported by Valenta et al. [27] in septic patients. In that research, the Se supplemented group showed an increasing trend of cholesterol levels from baseline to day 14 of treatment and its comparison with the control group showed significantly higher levels on day 14. They showed that Se restores cholesterol levels more efficiently. Future studies on the current topic are therefore needed.

## Oxidative stress in critical illness

Oxidative stress reflects an imbalance between the production of oxygen radicals and body antioxidant capacity. Moreover, oxidative stress through activating redox pathways for increased activation of transcription factors and inflammatory cytokines is one of the initiators of the inflammatory response [62]. Bulger et al. demonstrate that excessive oxidative stress may deteriorate the complications of critical illness, including multiple organ failure and ARDS [63]. It has been demonstrated that the levels of trace elements, including Se and zinc, may influence oxidative stress and response to inflammation in septic patients [54]. Since suboptimal Se levels have been reported in inducing oxidative stress, supplementation with trace elements, including Se, copper, and zinc, revealed beneficial effects on the improvement of infection following major burns [64]. On the other hand, oxidative damage per se can reduce Se and zinc levels and contribute to more oxidative stress [54].

### Se supplementation and oxidative stress markers

Since the reactive oxygen species have a short half-life, measuring byproducts of DNA, protein, and lipid oxidation can be used to determine the level of oxidative stress. Malondialdehyde (MDA) and Isoprostanes (IsoP) are among the valuable markers of oxidative stress and tissue damage which are the products of lipid peroxidation [65, 66]. MDA derives from attacks of free radicals on polyunsaturated long-chain fatty acids. This product is recently suggested as the most commonly used oxidative marker and can estimate or qualify oxidative stress at the early stages in critically ill septic patients [67]. The increased levels of MDA have been reported in pathological conditions, including septic patients or the ones at the risk of developing ARDS. Furthermore, its association with the severity of the inflammation has been demonstrated in critically ill patients [68]. The increased MDA may result from reduced free radical scavenging due to the altered status of trace elements in critical conditions such as major burns [69]. Since Se is present in antioxidant defensive systems, including erythrocyte GSH-Px and selenoenzymes, replacing this trace element might alleviate the oxidative stress-related complications of critical illness [70]. In this regard, Se supplemented-patients with a high risk of sepsis syndrome showed reduced levels of MDA starting at day three [71]. These results are consistent with those of other studies, which suggested the suppressive effects of Se supplementation on MDA levels in polycystic ovary syndrome (PCOS) and hemodialysis patients [72]. However, supplementation with sodium selenite (1000 µg per 2 h) increased MDA levels significantly by the fifth day of treatment, [31] which may be related to pro-oxidant effects of Se at high doses. Se supplementation in rats also revealed no changes in MDA levels [73].

F2-isoprostane (F2-isoP) is considered another useful biomarker of lipid peroxidation in various pathological conditions, such as critical illness, and can also reliably predict the oxidative response to different antioxidants [74, 75]. There is little research regarding the association of Se and F2-isoP levels or the effects of Se supplementary use. In one of these studies, Mishra et al. [49] showed no change in oxidative stress as measured by F2-isoP levels in the group supplemented with a high dose of Se compared to the group with a standard dose of Se, which suggests using other oxidative damage markers along with F2-isoP may give a more complete picture of oxidative stress.

### Se and antioxidant markers

The antioxidants stabilize the oxidant-antioxidant imbalance in favor of reducing oxidative damage and thereby decreasing the inflammatory response. Selenium, by incorporating into selenoproteins, is involved in one of the two antioxidant systems: the thiol redox system, including glutathione (GSH), glutaredoxin, GSH reductase, and GSH-Px and the thioredoxin (Trx) system, which consists of thioredoxin (Trx), Trx peroxidase and TrxR [76]. GSH-Px, a well-known free radical scavenger, detoxifies lipid hydrogen peroxide and hydroperoxides [77]. Alteration of GSH-Px levels is associated with the severity of oxidative damage in tissues [78]. Reduced levels of Se in parallel with the decreased GSH-Px selenoenzyme activity have been reported in SIRS and sepsis so that both markers are inversely correlated with the severity of clinical outcomes and may provide a predictive value for SIRS [15, 79]. Moreover, the overproduction of ROS and free radicals results in lung injury and increases the risk of critical illness in COVID-19. However, lung selenoproteins, by acting as antioxidants and modulating immune pathways, reduce virus invasion and lung injury [80]. Therefore, it can provide the rationale for Se supplementation in critically ill patients. In this regard, inorganic compounds of Se, administered intravenously or parenterally, enhanced the GSH-Px activity effectively in septic patients [81, 82]. Ebselen, an organoselenium compound, demonstrated antiviral activity via affecting the main protease of SARS-CoV-2 and its potential in mimicking GSH-Px and peroxiredoxin activates [83]. It has been reported that Se inhibits NF-kB, which is partially mediated by modulation of GSH-Px activity. Accordingly, inhibited NF-kB down-regulates pro-inflammatory genes and limits inflammatory response. On the other hand, Se can decrease the production of ROS and nitric oxide by modulation of p38 mitogen-activated protein kinase and NF-kB signaling pathways [84]. A positive and linear correlation was observed between serum Se and GSH-Px activity in critically ill patients. Moreover, Mahmoodpoor et al. reported a similar pattern between serum Se and GSH-Px following intravenous administration of sodium selenite in patients with sepsis and ARDS [51, 85]. Se supplementation in septic patients showed that the group received Se had significantly higher GSH-Px levels compared to those treated with placebo beginning at the first days of supplementation [86]. Furthermore, Se supplementation elevated GSH-Px-3 levels at days 3, 4, 7, and 10 of mechanical ventilation in patients with pneumonia [57, 85]. In agreement, another study has demonstrated that serum levels of GSH-Px were higher on day-7 and − 14 after Se supplementation in ARDS patients. These results match those reported in earlier studies [49, 87–89]. Surprisingly, Valenta et al. [27] showed that the increasing trend of GSH-Px within a week of supplementation with high-dose Se started to decrease after day-10, which is similar to the results found in a study by Manzanares et al. [90] The possible explanation for these results may be related to the insufficient synthesis of GSH and lack of precursors, including selenocysteine or hydrogen selenide.

The other components of the antioxidant defense system, including CAT and SOD are also involved in reducing ROS products [91]. However, Se supplementation in critically ill patients did not produce meaningful differences in the levels of SOD or CAT enzymes [31, 92]. Further research is required to determine the exact effect of Se on these antioxidant enzymes. However, based on the beneficial influence of Se administration on GSH-Px levels, it can be considered an effective micronutrient in restoring antioxidant capacity and preventing complications associated with a critical illness.

## Effects of Selenium on mortality rate and duration of ICU stay in critically ill patients

Kong L et al. reported that Se supplementation at doses higher than the daily requirement might decrease mortality in patients with sepsis. However, they declared that Se does not have any effect on the risk of nosocomial pneumonia or stay length in ICU [93]. Similarly, the results of one meta-analysis demonstrated that supplementing critically ill patients with Se reduced the overall mortality and resulted in a shorter length of hospital stay with no effect on 28-day mortality, stay length in ICU, the incidence of infection, and mechanical ventilation [94]. Designing more randomized clinical trials on this issue is essential to provide further evidence for clinical questions.

## Relationship between Covid-19 and the Selenim

About 5% of people with COVID-19 develop a severe illness marked by multiorgan dysfunction, systemic sepsis, and respiratory failure necessitating mechanical ventilation and ICU care [95, 96]. Such COVID-19 individuals experience pathological lung alterations [97]. Oxidative stress, which is caused by an excessive amount of free radical production in the lungs, is a significant contributor to the damage to the pulmonary tissue [98]. One of the most significant immunopathologic responses is thought to be caused by oxidative stress and lung lesions, which frequently develop into acute respiratory distress syndrome (ARDS). ARDS is also one of the most prevalent reasons of mortality in COVID-19 [99]. Selenium is crucial for severely sick COVID-19 patients, according to preliminary research [99, 100]. In addition, the severity of selenium insufficiency may be associated with a higher risk of death in critically sick patients. High dosages of selenium were discovered in a clinical experiment to lower septic shock mortality [101]. The most serious issues affecting severely sick COVID-19 patients are respiratory issues [101]. The impact of viral invasion and tissue damage can be lessened by lung selenoproteins, which also function as antioxidants and regulate a number of immune response pathways [102, 103].

## Conclusion

Selenium has anti-inflammatory and antioxidative effects and a reported link between the deficiency of Se and the severity of critical illness emphasizes the importance of this micronutrient. The benefits of Se therapy have been reported in clinical outcomes in patients with a critical illness. In recent years, the effects of seleno-compounds have been evaluated in ICU patients, particularly those with systemic inflammation and sepsis. The literature review showed the alteration of inflammatory markers, including procalcitonin, leukocyte count, albumin, prealbumin, CRP, inflammatory cytokines, and cholesterol following Se supplementation in critically ill patients. Besides, the antioxidant properties of Se due to its presence in the structure of several selenoenzymes have been reported. Various forms and dosages of Se supplementation have been used in different studies, mostly being the oral forms. Although most of them have all shown desirable effects in immunomodulation, using more uniform forms and dosages of this supplement can provide more conclusive results in the future. Meanwhile, the route of Se delivery, bolus or continuous administration, and the patient selection are different between studies and may influence the observed effects of supplementation. Furthermore, the blood levels of Se should be monitored to minimize the potential toxicity. Therefore, further studies are needed to establish clinical guidelines for Se supplementation in patients with a critical illness.
